# Trends of vasopressor using in medical intensive care unit: a 7-year cohort study

**DOI:** 10.1186/2197-425X-3-S1-A960

**Published:** 2015-10-01

**Authors:** N Srivali, C Thongprayoon, W Cheungpasitporn, K Kashani

**Affiliations:** Mayo Clinic, Rochester, United States

## Introduction

The use of vasopressor was common in medical intensive care unit (MICU). Due to the lack of conclusive evidence in superiority in efficacy among various types of vasopressors, the choice of vasopressor use mainly depends on the physician preference.

## Objectives

This study aims to describe the prevalence of vasopressor use and the trend in the use of each vasopressor medication in MICU over the past 7 years.

## Methods

This is a descriptive study conducted at a tertiary referral hospital. All MICU admissions at our institution between January 2007 and December 2013 were included in this study. The use of vasopressor within given ICU day (12.00 am - 11.59 pm) during ICU stay was reviewed. Vasopressors were defined as the continuous intravenous administration of norepinephrine, epinephrine, dopamine, phenylephrine, or vasopressin regardless of duration and dosage. The use of each vasopressor was reported as the vasopressor utilization index (VUI), using the following formula Vasopressor utilization index (VUI) = The total number of ICU days on a given vasopressor/The total number of ICU days on any vasopressor.

## Results

A total of 16,863 unique patients had 17,164 MICU admissions in the course of study, (55,391patient*ICU day).Out of 7,739 ICU days with vasopressor use, norepinephrine was used for 6,414 (83%), vasopressin for 1,960 (25%), phenylephrine for 772 (10%), dopamine for 623 (8%), and epinephrine for 323 (4%). From 2007 through 2013, there was an increasing trend in the use of norepinephrine (VUI_norepinephrine_ was 0.69 in 2007 and 0.91 in 2013) and an slight increasing trend in the use of epinephrine (VUI_epinephrine_ was 0.01 in 2007 and 0.06 in 2013). There was a decreasing trend in the use of dopamine (VUI_dopamine_ was 0.15 in 2007 and 0.03 in 2013), vasopressin (VUI_vasopressin_ was 0.42 in 2007 and 0.22 in 2013), and a slight downward trend in the use of phenylephrine.

## Conclusions

Norepinephrine is the most commonly used vasopressor in MICU. The use of norepinephrine and epinephrine are in upward trajectory.

**Figure 1:**
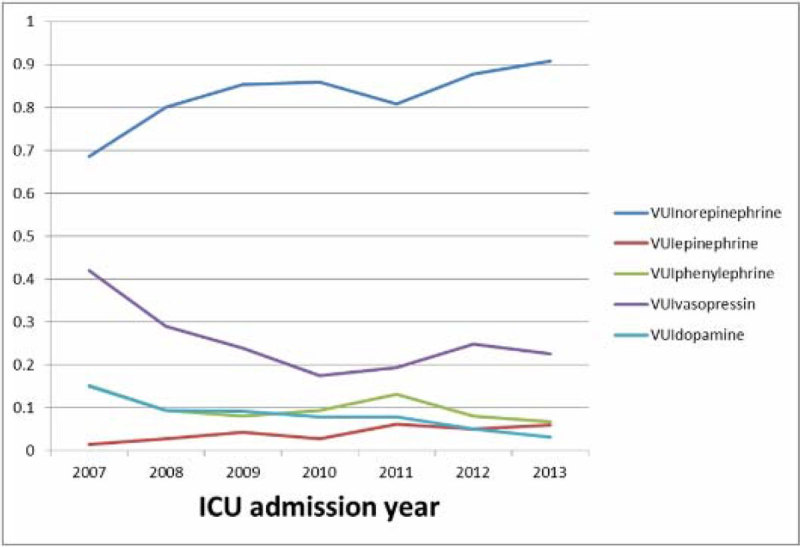
**Trend of vasopresser using**.
